# Delivery of Supported Self‐Management in Primary Care Asthma Reviews: Insights From the IMP^2^ART Programme

**DOI:** 10.1111/hex.70100

**Published:** 2024-11-12

**Authors:** Emma Kinley, Hilary Pinnock, Liz Steed, Kirstie McClatchey

**Affiliations:** ^1^ School of Psychology, Faculty of Health, Liverpool John Moore's University Liverpool UK; ^2^ Usher Institute The University of Edinburgh Edinburgh UK; ^3^ Wolfson Institute of Population Health Queen Mary University of London London UK; ^4^ NHS Tayside, Directorate of Public Health, Kings Cross Hospital Dundee UK; ^5^ School of Medicine, University of Dundee Dundee UK

**Keywords:** asthma, behaviour change, patient‐centred care, supported self‐management

## Abstract

**Background:**

Supported self‐management (SSM) for asthma reduces the risk of asthma attacks and improves asthma control and quality of life. SSM optimally includes patient‐centred communication and behaviour change support, however, the extent to which this occurs in routine primary care is unclear. This project was nested within the IMPlementing IMProved Asthma self‐management as RouTine (IMP^2^ART) programme; a UK‐wide trial evaluating an implementation strategy (including healthcare professional (HCP) training on behaviour change strategies and patient‐centred care) to improve support for asthma self‐management.

**Objective:**

To provide an understanding of how healthcare professionals deliver SSM in UK clinical practice; through assessing time spent on SSM strategies, how and to what extent patient‐centred care and behaviour change discussions are delivered, and to explore whether factors such as mode of review or implementation support influence delivery.

**Design, Setting and Participants:**

We conducted an observational study using video‐recordings of 12 HCPs delivering routine face‐to‐face and telephone asthma reviews (*n* = 64) in a sample of general practices participating in the IMP^2^ART trial (implementation *n* = 4; control *n* = 6). Analytical methods included: ALFA Toolkit Multi‐Channel Video Observation (to code and quantify tasks undertaken); the Patient‐Centred Observation Form; and The Behaviour Change Counselling Index (to assess patient‐centeredness and behaviour change counselling used by HCPs).

**Results:**

HCPs mostly spent time during routine asthma reviews discussing: an individual's asthma condition and management (average of 27.8% of consultation time); collaboratively reviewing and completing a personalised asthma action plan (6.3%) and training for practical self‐management activities (5.4%). Areas of patient‐centred care delivery and behavioural discussions included: creating and maintaining relationships, discussing asthma action plans and medication reconciliation. Professionals in IMP^2^ART implementation group practices delivered more SSM strategies. Comparison of face‐to‐face and remote consultations found no significant differences in HCP delivery of SSM.

**Conclusions:**

HCPs in UK primary care spent half the time in both face‐to‐face and remote asthma reviews delivering components of SSM suggesting that either mode of delivery may be acceptable. Reviews carried out in IMP^2^ART implementation group practices demonstrated more behaviour change and collaborative SSM strategies compared to those in the control group.

**Patient and Public Contribution:**

Asthma UK Centre for Applied Research (AUKCAR) PPI members were involved throughout, including project conception, providing feedback on participant‐facing documents, and discussing implications of study findings.

## 1 Introduction

Around 5.4 million people are currently receiving treatment for asthma, accounting for 2%–3% of primary care consultations and 60,000 hospital admissions annually [1, 2]. By controlling symptoms through safe and effective management and treatments, people living with asthma can reduce the risk of attacks. The management of asthma is an ongoing, dynamic process, where treatment and care needs to be continually reviewed and tailored to the individual's current needs and levels of severity.

Supported self‐management (SSM) is defined as an approach that includes both the healthcare professional (HCP) and the patient, and ensures patients with long‐term conditions have the knowledge, skills, confidence and support to manage the physical, emotional and social impact of their condition [3, 4]. SSM moves beyond a biomedical approach of patient‐provider communication, where patients are provided with information or treatment options, to a biopsychosocial, healthcare professional and patient relationship, where patients can play a joint role in guiding their own care [5]. Recommendations for the clinical and personal benefits of implementing SSM for asthma have been proposed since the early 1990s [6, 7]. Since then, national and international guidelines [8, 9, 10] have recommended that people with asthma should be provided with self‐management education, reinforced by an asthma action plan and supported by regular review [11, 12].

The effectiveness of SSM for asthma has been demonstrated in a range of populations including diverse cultural groups [13], children [14] and adults [11, 15]. Despite the ‘overwhelming’ evidence of effectiveness [12], SSM is not yet embedded in routine clinical practice [16]. Implementation of asthma self‐management in routine care can be achieved, but it is argued that to be effective, it requires a whole‐systems approach, which considers patient education and resources, healthcare professional skills, and motivational and organisational priorities and routines [17].

Healthcare professionals are ideally placed to support self‐management and facilitate behaviour change because of their regular one‐to‐one patient contact [18], but the effectiveness of this interaction is influenced by the behaviours of both the patient, healthcare provider, and the relationship between the parties [19]. Within primary care, effective patient‐centred communication in clinical interactions can enhance patient engagement in decision making, improve patient adherence to medication and treatment plans, increase social support, safety, and patient satisfaction in care [20, 21].

Remote consulting, already promoted as a partial solution to growing challenges of healthcare delivery, was introduced rapidly in response to the global coronavirus disease 2019 (COVID‐19) pandemic to avoid face‐to‐face contact and thereby minimise infection risk to patients and healthcare staff [22]. Remote consultations are now routine, actively chosen by some patients for delivery of care, as opposed to a public health necessity [23]. Different forms of clinical consultations have advantages and disadvantages for different individuals and patient groups [24], and there is a need for evidence exploring the impact remote asthma consultations have upon a patient's care and ability to self‐manage.

The IMP^2^ART (IMPlementing IMProved Asthma Self‐Management as RouTine) trial [25, 26] aims to improve implementation of supported asthma self‐management in UK primary care by delivering a theoretically informed whole‐systems implementation strategy comprising of;

- resources for patients (e.g., an asthma information website),
- professional training for primary care staff and HCPs including a facilitator‐led workshop and two online educational modules. One module for all primary care staff raised awareness of the benefits of SSM and aimed to increase engagement, motivation and commitment to supporting self‐management as a priority across the whole practice team. The second module aimed to enable individual HCPs to use behaviour change strategies in clinical practice to deliver effective SSM) and
- organisational components (e.g., audit and feedback reports) [25].

### 1.1 Aims

There is a gap in the current literature on how asthma reviews are actually delivered in practice. This is important given knowledge that there is often a guideline‐implementation gap and hence actual practice cannot be assumed. We provide an observational understanding of how SSM is routinely delivered in UK primary care. We explored how SSM is delivered by healthcare professionals during routine primary care asthma reviews as part of the IMP^2^ART trial.

We aimed to assess if/how healthcare professionals are using patient‐centred and motivational strategies to promote asthma self‐management. Our primary objective was to observe the time allocated to self‐management tasks within routine reviews, and explore how patient‐centred methods and behaviour change counselling are used within asthma consultations. Sub‐group analyses explored differences in relation to allocation in the IMP^2^ART trial (implementation or control) and mode of delivery (remote telephone or face‐to‐face consultations).

## 2 Methods

### 2.1 Design

This observational research was conducted between November 2021 and July 2022 with ethical approval from by NHS Lothian Research Ethics Committee (REC) (Ref No: 20/NI/0177). During this time, remote reviews were a regular mode of asthma consultation due to the COVID‐19 pandemic, although face‐to‐face reviews were being reinstated as restrictions eased. Comparisons were made between delivery of SSM in face‐to‐face and remote modes of consultations and during routine asthma reviews by healthcare professional in the IMP^2^ART trial implementation and control groups.

### 2.2 Participants

For the purposes of this study, the healthcare professional was the member of staff in the practice who regularly conducted asthma reviews (e.g., practice nurse or clinical pharmacist). Healthcare professionals were only recruited from participating IMP^2^ART practices (implementation or control groups) and must have attended the IMP^2^ART facilitation workshop (for implementation group professionals only).

Patients with asthma recruited to the study had an asthma diagnosis for which medication had been prescribed in the last 12 months, were scheduled for an asthma review within the participating practice (may have other co‐morbidities but primary reason for appointment was an asthma review) and were aged 5 years and over. Patients with asthma must have been able to give consent to participate in the study and able to understand and communicate in the English language.

### 2.3 Recruitment and Procedure

Healthcare professionals were identified from general practices participating in the IMP^2^ART trial (either implementation or control practices). Potentially interested professionals were contacted and a meeting arranged to discuss the details of study and to take written informed consent. Arrangements were then made for ‘recording‐clinics’. These clinics were organised specifically for patients who had consented to take part in the research. Each individual asthma review was recorded by video (if taking place face‐to‐face in the consultation room) and audio recorded (if conducted via telephone).

Patients with an annual asthma review scheduled for the ‘recording clinic’ were approached by the participating HCP and informed of the research using information and consent documents. In line with standard consent procedures, parents/guardians of patients under the age of 16 were asked to complete consent on children/young person's behalf. All information and consent documents were designed with the advice of the Patient and Public Involvement (PPI) colleagues from Asthma UK Centre for Applied Research (AUKCAR).

Patients wishing to take part provided written consent for their asthma review to be recorded, viewed, and analysed. Patients were provided with study information and consent at least 2 weeks before recordings took place and were free to withdraw at any time.

### 2.4 Data Handling

Video and audio recordings of routine asthma reviews were collected on an encrypted video/audio recorder, stored on an encrypted laptop then transferred onto an encrypted University of Edinburgh Data Store server. Data were transferred from the camera using a secure data sync service. All files were encrypted using ‘VeraCrypt container encryption’ service which is password protected. Only the research team had permissions to access data storage systems.

### 2.5 Measures

E.K., who as Health Psychologist has expertise in understanding the psychological aspects of health and illness and behaviour change techniques, used three tools to code the collected video/audio data.

### 2.6 Activity Log Files Aggregation (ALFA) Toolkit

The ALFA Toolkit [27], a framework for describing common parts of a primary healthcare consultation, was used to measure the time taken on SSM tasks during routine asthma reviews (Supporting Information: Appendix 1). As there is no verified tool that specifically explores the delivery of SSM, the components of the PRISMS (Practical Reviews in Self‐Management Support) [28] were incorporated within the ALFA toolkit to measure time taken on specific SSM tasks. The adapted ALFA measure included 18 items, for example: *‘Time spent discussing individual asthma condition and/or its management’*, *‘Time spent discussing asthma control and possible triggers, practical support with adherence (medication or behavioural)’* and *‘Time spent discussing lifestyle factors e.g., smoking, diet, exercise’*. Only SSM tasks were coded throughout the interaction. All other tasks or time spent in silence were not coded.

### 2.7 Patient‐Centred Observation Form (PCOF)

The PCOF [29] is a communication and relationship assessment tool used to measure healthcare professional levels of patient‐centred communication. The 13 category, 53 item‐measure grades the healthcare professional on the level of biomedical or biopsychosocial communication in the consultation (depending on usage of elements) specific to each item (e.g., *‘uses eye contact’*, *‘uses verbal or non‐verbal empathy’, ‘asks if a patient wants to create a health goal’*). A higher mean PCOF score (closer to the possible maximum score for PCOF category) indicates healthcare professionals with a more patient‐centred and biopsychosocial focus during delivery of care. Lower mean PCOF category scores indicate lower patient‐centred care delivery, and a more biomedical focus. Reviews were coded out of 53, one for each element that was evident within the recorded asthma review.

### 2.8 Behaviour Change Counselling Index (BECCI)

The BECCI [30] is a tool used to measure practitioner competence in behaviour change counselling skills during healthcare consultations. The 11 items are scored using a 5‐point Likert scale. Mean total scores, also known as Practitioner BECCI scores, are calculated as the mean across all items, ranging from 0 (not at all) to 4 (a great extent). Example items included in the checklist are: *‘Practitioner invites the patient to talk about behaviour change’* and ‘*Practitioner actively conveys respect for patient choice about behaviour change.’*

### 2.9 Statistical Analysis

The statistical analyses for this study were performed using the IBM SPSS statistics software (Version 25) [31]. To provide a summary of the sample characteristics and main variables of interest, descriptive statistics were calculated, including mean and standard deviations to describe averages of variables. The determinant of sample size was considered by reaching data saturation from the in‐depth style of data collection.

Duration of the reviews, and the ALFA, BECCI and PCOF scores were compared between HCPs within IMP^2^ART implementation and control groups, and differences between HCPs delivering face‐to‐face and remote asthma reviews using independent sample *t*‐tests or non‐parametric tests (Mann–Whitney tests) depending on levels of distribution.

To explore correlation between the three tools used to measure the delivery of SSM for healthcare professionals (ALFA, PCOF and BECCI), a correlation matrix was produced. The matrix shows the correlation coefficients and p‐values for levels of statistical significance and correlation between variables.

## 3 Results

### 3.1 Characteristics of Practices and Participating Professionals

Recordings of routine asthma reviews were collected from 10 (*n* = 6 control; *n* = 4 implementation) IMP^2^ART general practices from across the UK. Twelve healthcare professionals (from the 10 general practices) participated in the study. Sixty‐four (control, *n* = 37; implementation, *n* = 27) unique patient asthma reviews were recorded (both face‐to‐face, *n* = 49, and remote telephone reviews, *n* = 15). See Table 1 for details.

**Table 1:** Overview of collected observational recorded data.

Data characteristics	Overview of collected data
Total number of recruited general practices	10 general practices: 4 IMP^2^ART implementation and 6 IMP^2^ART control practices
Total number of primary care practitioners	12 healthcare professionals: 11 practice nurses and 1 clinical pharmacist 4 IMP^2^ART implementation healthcare professionals and 8 control healthcare professionals
Total number of asthma reviews recorded	64 routine primary care asthma reviews: 49 face‐to‐face and 15 remote telephone consultations 27 from the IMP^2^ART implementation group and 37 from IMP^2^ART control group
Duration of asthma reviews	Asthma reviews averaged 16 min 51 s. Range from 6 min to 47 min

Abbreviation: IMP^2^ART, IMPlementing IMProved Asthma self‐management as RouTine.

Before the observations, all healthcare professionals in the implementation practices had completed the two IMP^2^ART education modules. Demographic details of each recruited practice, including practice location, asthma patient list size, deprivation score and amount of time implementation strategy had been embedded into routine care, are detailed in Table 2.

**Table 2:** Demographic details of recruited practices, in chronological order of data collection visit.

Practice No.	IMP^2^ART implementation/control group	No. of recruited HCPs	Practice clinical research network location	Practice list size (grouped)	Asthma patient list size	GP deprivation score^a^	Amount of time allocated for asthma review	Time since allocation to implementation
1	Control	2 (practice nurses)	Kent, Surrey and Sussex	> 20,000	2919	14.5	20 min	*—*
2	Implementation	1 (practice nurse)	Yorkshire and Humber	10,000–20,000	855	31.2	30 min	6 months
3	Implementation	1 (practice nurse)	Northwest Coast	10,000–20,000	600	32.4	20 min	9 months
4	Control	1 (clinical pharmacist)	Wessex	< 10,000	492	16.3	10–30 min	*—*
5	Control	1 (practice nurse)	Yorkshire and Humber	10,000–20,000	1206	31.3	20 min	*—*
6	Control	1 (practice nurse)	West of England	< 10,000	570	27.2	20 min	*—*
7	Implementation	1 (practice nurse)	Kent, Surrey and Sussex	< 10,000	448	11.5	15–20 min	3 months
8	Control	2 (practice nurses)	East of England	< 10,000	657	27.2	20 min	*—*
9	Control	1 (practice nurse)	NHS Research Scotland South	< 10,000	679	2.1^b^	30 min	*—*
10	Implementation	1 (practice nurse)	North Thames	< 10,000	439	19.3	20 min	5 months

Abbreviations: GP, general practitioner; HCPs, healthcare professionals; IMP^2^ART, IMPlementing IMProved Asthma self‐management as RouTine.

a Deprivation status of practice: (England median = 21.4 [ ≤ 21.4 is less deprived; > 21.4 is more deprived]) (Scotland median = 3.1 [ ≤ 3.1 is less deprived; > 3.1 is more deprived]).

b Scottish deprivation score is not comparable to English practices.

#### 3.1.1 Proportion of the Review Addressing SSM Tasks (ALFA Scores)

**Figure 1:**
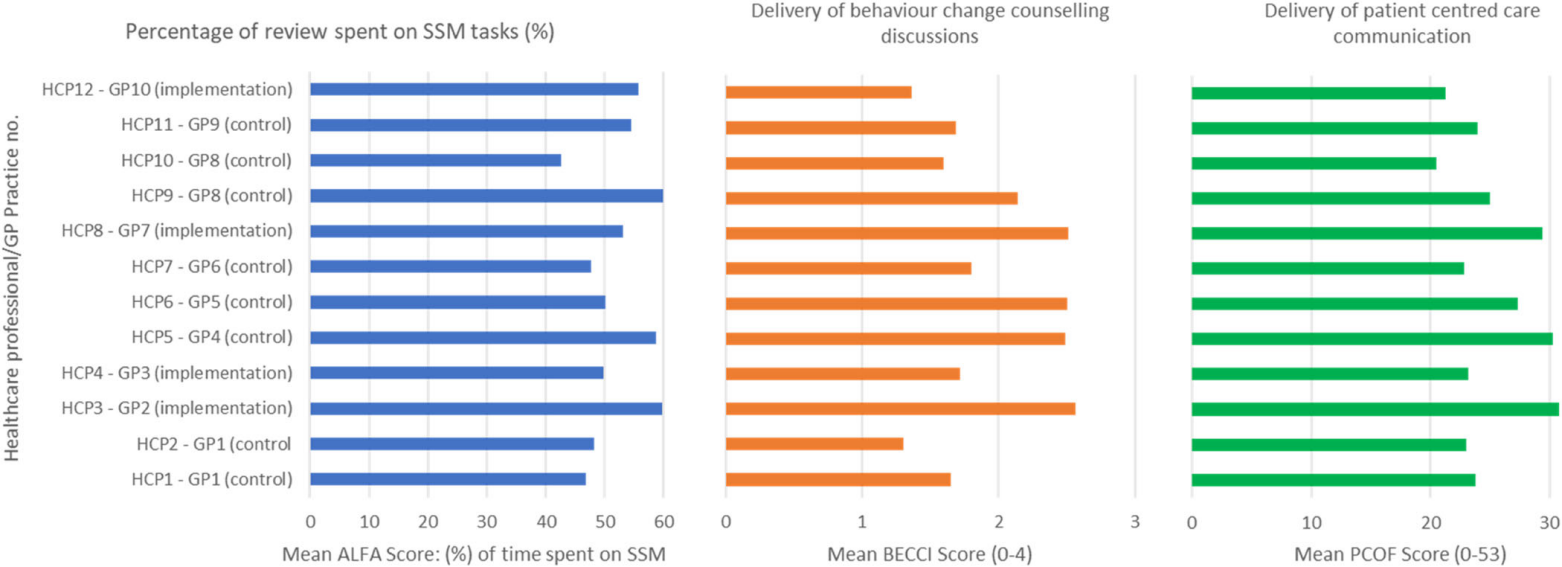
Mean ALFA (proportion of asthma reviews spent on PRISMS‐defined self‐management tasks), BECCI scores (delivery of behaviour change counselling discussions) and PCOF (delivery of patient‐centred care communication) per HCP and per general practice. ALFA, Activity Log Files Aggregation; BECCI, Behaviour Change Counselling Index; HCP, healthcare professional; PCOF, Patient‐Centred Observation Form; PRISMS, Practical Reviews in Self‐Management Support; SSM, self‐management support.

In the 64 video‐recorded asthma reviews, on average, healthcare professionals spent 53.1% (SD 4.92%; range 35.65%–69.56%) of the consultation time on tasks related to SSM. Table 3 shows the PRISMS‐defined self‐management components [28] and the mean proportion of time spent on each component during the recorded asthma reviews for all HCPs. The SSM task on which healthcare professionals spent the most time was *‘A1: Time spent discussing individual asthma condition and/or its management’* with an average percentage of time of 27.8% (SD = 7.7%). The mean proportion of reviews spent on PRISMS‐defined self‐management tasks per healthcare professional and general practice is shown below in Figure 1.

**Table 3:** Average proportion of the asthma review time spent on PRISMS‐defined self‐management components [28].

Supported self‐management component as defined in PRISMS taxonomy [25]	Mean % (SD) of time spent on component during total recorded asthma reviews	Range of % of time spent on component during recorded asthma reviews
Time spent discussing individual asthma condition and/or its management (A1)	27.8% (7.74)	13.8%–43.9%
Time spent referring to other available asthma resources and services (improving access to services) (A2)	2.7% (2.14)	0.2–7.4%
Time spent collaboratively reviewing and completing personalised asthma action plan (A3)	6.3% (5.82)	0.7%–19.8%
Time spent discussing attendance to regular reviews (A4)	1.1% (0.56)	0.5%–2.3%
Time spent providing feedback on individual monitored asthma data (A5)	5.8% (4.87)	0.7%–18.2%
Time spent discussing asthma control and possible triggers, practical support with adherence (medication or behavioural) (A6)	3.6% (2.77)	0.4%–14.6%
Time spent providing equipment/discussion of new equipment (A7)	3.4% (3.92)	0.6%–18.1%
Time spent discussing how to access advice or support when needed (A8)	1.4% (0.81)	0.4%–3.1%
Time spent training/rehearsal to communicate with health care professionals (A9)	1.4% (0.28)	1.4%–1.4%
Time spent on training/rehearsal for everyday activities (A10)	2.3% (1.77)	0.3%–6.8%
Time spent training/rehearsal for practical self‐management activities (e.g., inhaler technique) (A11)	5.4% (3.76)	0.5%–15.6%
Time discussing psychological strategies (problem solving, goal setting, action planning, relaxation techniques etc). (A12)	4% (3.62)	1%–11.5%
Time discussing individual Social Support (A13)	2.7% (1.22)	1.2%–3%
Time spent discussing lifestyle factors e.g., smoking, diet, exercise. (A14)	4.4% (4.02)	0.3%–15.8%
Other: Time spent on screen sharing (discussing something on screen with the patient) (A15)	6% (7.47)	0.7%–11.3%
Other: time spent setting the patient agenda for the consultation (A16)	1% (0.75)	0.4%–2.6%
Other: time spent talking about other multimorbidities (A17)	3.8% (2.36)	0.4%–8.9%

Abbreviations: PRISMS, Practical Reviews in Self‐Management Support; SD, standard deviation.

#### 3.1.2 Delivery of Patient Centred Care Communication (PCOF)

Of the 64 recorded asthma reviews, healthcare professionals scored an average of 26 (out of a possible 53) and scores ranged from 18 to 36. Mean scores for each PCOF grouping can be seen in Table 4; mean PCOF scores for all individual HCPs are shown in Figure 1.

**Table 4:** Mean PCOF grouping scores of asthma reviews for all HCPs.

PCOF grouping^a^	PCOF score mean (SD)	Range of PCOF score
Establishes rapport	4.6 (0.71)	2–5
Maintaining relationship through the interaction	3.7 (0.61)	2–4
Collaborative upfront agenda setting	0.6 (0.66)	0–3
Maintains efficiency through thinking out loud	1.31 (0.92)	0–4
Basics: Vitals, checks meds and paperwork	2.9 (0.78)	1–4
Patient activation and engagement	2.8 (1.32)	0–5
Electronic medical record use	1.8 (1.09)	0–4
Gathering information	2.9 (0.3)	1–3
Self‐management support: goal setting and action plan development	1.6 (1.7)	0–7
Self‐management follow‐up: Checking on progress, revision	2.1 (1.02)	0–4
Closure and system navigation	1.78 (1.06)	0–4

Abbreviations: HCP, healthcare professional; PCOF, Patient‐Centred Observation Form; SD, standard deviation.

a PCOF grouping scores: the higher mean PCOF score indicated that healthcare professionals have a more patient‐centred and biopsychosocial focus during delivery of care. Lower mean PCOF grouping scores indicated lower patient‐centred care delivery, and a more biomedical focus.

#### 3.1.3 Delivery of Behaviour Change Discussions (BECCI)

Of the 64 asthma reviews, healthcare professionals scored an average of 2 out of 4 for BECCI scores, indicating on average, healthcare professionals delivered behaviour change discussions *‘to some extent’*. Mean scores for each BECCI item can be seen in Table 5. Mean BECCI scores for all healthcare professionals were calculated for each BECCI item, shown below in Figure 1.

**Table 5:** Average BECCI item scores of asthma reviews.

BECCI Item	BECCI score mean (SD)	Range of BECCI score
1. Practitioner invites the patient to talk about behaviour change	1.8 (0.85)	0–4
2. Practitioner demonstrates sensitivity talking about other issues	2.5 (0.8)	1–4
3. Practitioner encourages patient to talk about current behaviour or status quo	2.2 (0.73)	1–4
4. Practitioner encourages patient to talk about change	1.7 (0.83)	0–4
5. Practitioner asks questions to elicit how patient thinks and feels about the topic	1.6 (0.71)	0–3
6. Practitioner uses empathic listening statements when the patient talks about the topic	2.4 (0.77)	1–4
7. Practitioner uses summaries to bring together what the patient says about the topic	2.4 (0.5)	2–3
8. Practitioner acknowledges challenges about behaviour change that the patient faces	1.5 (0.91)	0–4
9. When practitioner provides information, it is sensitive to patient concerns and understanding	2.6 (0.64)	1–4
10. Practitioner actively conveys respect for patient choice about behaviour change	2.1 (0.77)	1–4
11. Practitioner and patient exchange ideas about how the patient could change current behaviour	1.4 (0.99)	0–4

Abbreviations: BECCI, Behaviour Change Counselling Index; SD, standard deviation.

### 3.2 Subgroup Analyses

Subgroup analysis of allocation in the IMP^2^ART trial (implementation or control), mode of delivery (remote telephone consultations or face‐to‐face) in relation to each measure, duration of asthma reviews and descriptive mean scores for each measure are provided in Table 6.

**Table 6:** Subgroup analysis of duration of review, ALFA, PCOF and BECCI scores per grouping.

	No.	Mean (SD)	Mean difference (95% CI)	*p*‐Value
Duration of review (in minutes)				
All reviews	64	16.9 min (7.6)		
IMP^2^ART Implementation group	27	16.8 min (7.5)	−2.1 [95% CI −6.30 to 2.07]	*p* = 0.691
IMP^2^ART control group	37	18.6 min (9.3)		
Face‐to‐face review	49	18.8 min (8.6)	5.88 [95% CI 1.19–10.58]	*p* = 0.026
Telephone review	15	12.9 min (4.9)		
Proportion of review on supporting self‐management (ALFA) (% of overall review time)				
All reviews	64	53.1 (4.9)		
IMP^2^ART Implementation group	27	55.3 (7.8)	4.4, [95% CI 0.3–8.5],	*p* = 0.038
IMP^2^ART control group	37	50.9 (8.4)		
Face‐to‐face review	49	52.8 (8.2)	0.0, [95% CI −5.0 to 5.0]	*p* = 0.993
Telephone review	15	52.8 (9.2)		
Delivery of patient‐centred care (PCOF score)				
All reviews	64	26.0 (5.01)		
IMP^2^ART Implementation group	27	27.6 (5.61)	2.5, [95% CI 0.7–4.9]	*p* = 0.044
IMP^2^ART control group	37	25.1 (4.11)		
Face‐to‐face review	49	26.3 (4.76)	0.8, [95% CI −2.2 to 3.7]	*p* = 0.608
Telephone review	15	25.5 (5.55)		
Delivery of behaviour change discussions (BECCI score)				
All reviews	64	2.0 (0.57)		
IMP^2^ART Implementation group	27	2.2 (0.58)	*0.3, [95% CI 0.0–0.6]	*p* = 0.038
IMP^2^ART control group	37	1.9 (0.52)		
Face‐to‐face review	49	2.2 (0.53)	0.3, [95% CI −0.0 to 6.4]	*p* = 0.055
Telephone review	15	1.8 (0.63)		

*Note:* Unless indicated statistical analyses uses independent sample t‐tests.

Abbreviations: ALFA, Activity Log Files Aggregation; BECCI, Behaviour Change Counselling Index; CI, confidence interval; IMP^2^ART, IMPlementing IMProved Asthma self‐management as RouTine; PCOF, Patient‐Centred Observation Form; SD, standard deviation.

^a^ Mann–Whitney *U*.

#### 3.2.1 Duration of Review and Proportion Used for Self‐Management Tasks

On average, healthcare professionals spent 16.9 min delivering a routine asthma review. Remote telephone consultations were significantly shorter than face‐to‐face reviews (12.9 min vs. 18.8 min), but a similar proportion of the reviews were used for self‐management related tasks. There was no difference in duration of review between IMP^2^ART implementation and control practices, but practitioners in IMP^2^ART implementation group practices spent a significantly greater proportion of the review on SSM tasks (55.3% vs. 50.9%).

#### 3.2.2 PCOF and BECCI

Compared to control group practices, healthcare professionals in IMP^2^ART implementation group practices delivered care that was more patient‐centred (PCOF score 27.6 vs. 25.1 (SD = 4.1) and included more behaviour change counselling discussions (BECCI score: 2.2 vs. 1.90). In contrast, the PCOF and BECCI scores in remote and face‐to‐face consultations showed no significant differences.

#### 3.2.3 Correlation Matrix

The correlation matrix table (provided in Table 7) shows the correlation matrix, displaying the correlation coefficients and *p* values for levels of statistical significance between variables. All three measures are linearly related. The highest correlation level was between the PCOF and BECCI scores (*r* = 0.74**, *p* = *0.001*), which measure the delivery of patient‐centred care and behaviour change counselling discussions.

**Table 7:** Correlation Matrix of correlations between ALFA, PCOF and BECCI variables.

	ALFA scores	BECCI scores	PCOF scores
ALFA scores			
BECCI scores	*r* = 0.41**, *p* = *0.001*.		
PCOF scores	*r* = 0.45**, *p* = *0.001*.	*r* = 0.74**, *p* = *0.001*.	

Abbreviations: ALFA, Activity Log Files Aggregation; BECCI, Behaviour Change Counselling Index; PCOF, Patient‐Centred Observation Form.

** Correlation is significant at the 01 level (two‐tailed).

## 4 Discussion

### 4.1 Summary of Study Findings

The findings of this observational study in UK primary care provide insights into the way SSM is delivered in clinical practice, including understanding time spent on SSM strategies and how patient‐centred and behavioural discussions are delivered. Healthcare professionals (most commonly practice nurses) spend an average of 16 minutes delivering a routine asthma review, about half of which is used to address tasks related to SSM, though there was a substantial diversity between practices and individual practitioners. Discussing the individual's asthma condition and/or its management, collaboratively reviewing and completing a personalised asthma action plan, training/rehearsal for practical self‐management activities (e.g., inhaler technique) and screen sharing were the commonest tasks undertaken.

On average, healthcare professionals delivered around half of the patient‐centred care components (as measured by the PCOF). Higher levels of patient‐centred care (indicating a biopsychosocial focus of delivery of care) were in areas such as establishing rapport, maintaining relationships throughout the interaction, and gathering information such as vitals, medications and paperwork. Lower levels of patient‐centred communication (indicating a biomedical focus) were found in collaborative upfront agenda setting, self‐management support (including goal setting and action plan development), and closure and system navigation aid support. Healthcare professionals delivered behaviour change counselling discussions *‘to some extent’*. BECCI items where healthcare professionals discussed behaviour change ‘*a good deal’* included; demonstrating sensitivity talking about patient concerns and understanding and when providing information. Items where healthcare professionals delivered behaviour change discussions *‘minimally’* included; acknowledging challenges about behaviour change that the patient faces and exchanging ideas about how the patient could change current behaviour.

IMP^2^ART implementation groups, on average, spent more time during the consultation incorporating and discussing SSM strategies during routine reviews, delivered a more patient‐centred review and had more behaviour change discussions, compared to healthcare professionals of the IMP^2^ART control group. Healthcare professional delivery of SSM between face‐to‐face and remote consultations showed no significant differences for percentages of time on SSM tasks during routine asthma reviews and delivery of patient‐centred communication and behaviour change discussions.

### 4.2 Discussion of Findings in Relation to Existing Literature

It is recommended that primary care asthma reviews in the UK should be allocated between 20 and 30 min [32]. However, we observed that healthcare professionals spent 16 min (on average) delivering a routine appointment. Issues affecting time allocated to routine reviews include staff shortages due to ill health, burnout and workforce issues in an already pressured system [33]. Healthcare professionals therefore need to evolve efficient strategies to incorporate and discuss meaningful SSM strategies during time‐limited asthma reviews.

It has been argued that implementation of asthma self‐management in routine reviews can be achieved, but to be effective, it requires a whole‐systems approach, which considers patient education and resources, healthcare professional skills and motivational and organisational priorities and routines [16, 28]. IMP^2^ART implementation practices in our study were associated with a more patient‐centred review and more behaviour change discussions, despite similar duration of the consultation. Previous research [17] has suggested there is a gap in healthcare professional education in relation to use of theoretical frameworks or evidence‐based structures to implement SSM and facilitate patient behaviour change in asthma reviews. The current findings suggest theory informed healthcare professional training can influence the delivery of SSM and may increase healthcare professional's implementation [25], hence addressing the identified need.

Previous research has also raised concerns about the use of remote delivery of routine primary care due to concerns about suitability and the associated technical, clinical, and organisational policy challenges [34]. More specifically, research regarding the delivery of SSM during remote asthma consultations has rapidly dated with advances in technology [34, 35]. This study adds to prior knowledge as it was undertaken immediately post‐COVID, so comparisons were able to be made between healthcare professionals' delivery of SSM, communication styles and behaviours within the changing context for remote and face‐to‐face asthma consultations. HCPs spent similar amounts of time delivering SSM strategies, providing patient‐centred care and discussing behaviour change in face‐to‐face and remote consultation groups. This clarifies that remote consultations may be a useful alternative to see specific groups of patients (e.g., those with well‐controlled asthma and who have an existing relationship with their primary healthcare professional [36]. However, healthcare professionals need to be aware that people living with asthma may still value the interpersonal, holistic communication opportunities associated with face‐to‐face care. The findings of this study are consistent with existing research that healthcare professionals should use advanced communication skills to identify patient's views and preferences to arrive at shared goals and plans [21].

### 4.3 Strengths and Limitations

The study has several strengths. The high level of correlation between the quantitative tools used lends credibility to the findings. This correlation analysis provides evidence that all three measures have an overlap and are linearly related, which suggests use of all three measures together may be an effective tool to observe the delivery of SSM, including delivery of patient‐centred care and behaviour change counselling discussions. Alternatively, this outcome could also suggest one measure could be used to measure SSM delivery, due to the strong correlation. Another strength of this study was the recording of real‐world asthma reviews as opposed to simulated scenarios, which increases the applicability of the findings. A major strength of this research is the partnership with the AUKCAR PPI colleagues. PPI members were involved throughout the whole project and all feedback was acknowledged, valued, and relevant updates were made. Further, results were formally presented to a group of PPI members who provided feedback and suggested possible implications for those living with asthma, especially noting the importance of patient choice during remote asthma reviews.

The generalisability of these results are subject to certain limitations. The Hawthorne effect [37] (where individuals who are being recorded may alter their behaviour in response to their awareness of being observed) may have had an impact. However, this would have been apparent across both the implementation and control groups and during remote of face‐to‐face reviews and therefore should not impact on the sub‐group analyses in this study. Similarly, the personality, confidence, and expertise/skills of the healthcare professional may have affected the level of engagement, asthma management and SSM strategies discussed whilst being recorded. Within the HCP sample, there may have been a sampling bias, where more confident healthcare professionals agreed to participate. However, there were varying levels of healthcare professionals' expertise and skills.

HCPs who took part in the recordings, may have scheduled longer asthma reviews than their usual appointments, as information from the practices stated that they usually only have 20 min allocated. Practices had organised an afternoon or morning of scheduled recordings for the ‘recording clinic’, sometimes lasting up to 30 min per patient, which may not reflect how asthma reviews are normally delivered. This may have led to HCPs discussing SSM components in greater detail due to longer scheduled routine reviews. An additional factor was that patient participation was influenced by the HCP, as the recorded clinics were set‐up in advance. This approach may have allowed healthcare professionals to select participants with more well‐controlled asthma who they considered were more effective with their self‐management strategies. This obstacle was difficult to overcome due to the ethical requirements of provide information in advance and obtaining informed consent, though this would have been the case across the implementation and control groups, limiting the impact for any subgroup analysis.

### 4.4 Implications for Clinical Practice and Research

The encouraging findings associated with the IMP^2^ART implementation strategy on healthcare professional communication, behaviour and SSM delivery, suggest that healthcare professionals should be provided with specific training in SSM delivery (including the incorporation of the IMP^2^ART implementation strategies). The training should be developed and embedded with the theoretical underpinnings of patient‐centred communication, shared decision making, and behaviour change theories and models to increase effectiveness.

We have identified areas of improvement for HCP communication and SSM delivery, including increased scope to have behavioural change discussions and upfront agenda setting. Primary care conversations are often missed opportunities for promoting good health and wellbeing in general practice. Healthcare professionals should view each individual patient, and each individual contact with a patient as an opportunity for health improvement [21, 38]. Asthma consultations include opportunities for healthcare professionals to engage in behaviour change discussions (e.g., engaging in exercise to improve lung health, quitting smoking etc.). However, evidence‐informed training is needed to improve the capability and confidence of healthcare professionals to have behaviour change discussions in primary care [21]. Our findings suggest that HCPs are delivering empathetic conversations and care to patients but are not collaboratively discussing individualised approaches for ways in which a patient can proactively change their behaviour, which has also been identified in recent research assessing competence of respiratory healthcare professionals to deliver a psychologically based interventions [39]. Training (to increase knowledge, skills and confidence) and resources could improve behavioural discussions during asthma consultations. Future research should explore HCPs delivery of behaviour change communication within primary care asthma reviews, to create a clearer picture of resources and training needed for HCPs to implement a consistent approach.

Remote asthma reviews (synchronous communications which include real‐time telephone consultations) were found to be similar in how SSM is delivered compared to face‐to‐face reviews. However, policy makers should consider the specific groups of asthma patients who may benefit from remote technologies and be aware of the limitations remote tools may have on some people with asthma (e.g., access to remote devices, patient literacy levels, and the risk of increasing socioeconomic inequalities). Consequently, remote asthma reviews should be provided according to the preference of the patient, and therefore coinciding with the patient‐centred nature of primary care reviews, patients and professionals should work together to reach an agreed decision about the mode of delivery for a patient's care [1, 24].

The implementation of SSM strategies for asthma is multifaceted and complex. Further components of implementation e.g., quality, sustainability, and patients' feedback are due to be explored within the wider IMP^2^ART programme of work [25, 26].

## 5 Conclusions

This observational research has provided an overview of how SSM is delivered in UK primary care practice. The HCPs in our study spent substantial proportions of asthma reviews addressing tasks related to SSM. Professional training can build on these encouraging findings to prepare HCPs to deliver patient‐centred care and SSM in routine practice. Participation in the theoretically informed IMP^2^ART implementation strategy was associated with increased time spent on SSM tasks and delivery of patient‐centred care and behavioural discussions, potentially to benefit of people with asthma. Routine remote reviews may be an acceptable alternative to deliver SSM for asthma care for specific patient groups.

## Author Contributions

**Emma Kinley:** conceptualisation, writing–original draft, investigation, methodology, writing–review and editing, software, formal analysis, project administration, data curation, resources, funding acquisition, validation, visualisation. **Hilary Pinnock:** supervision, conceptualisation, writing–review and editing, methodology, project administration, validation, visualisation. **Liz Steed:** supervision, conceptualisation, writing–review and editing, methodology, project administration, validation, visualisation. **Kirstie McClatchey:** supervision, conceptualisation, writing–review and editing, methodology, project administration, validation, visualisation.

## Ethics Statement

Ethical approval was granted by NHS Lothian Research Ethics Committee (REC) (Ref No: 20/NI/0177) and regulatory approvals were approved and sought from the Health Research Authority (Ref No: 20/NI/0177) and National Research Scotland (Ref No: NRS21/280392). Both healthcare professionals and patients with asthma provided written informed consent before involvement.

## Conflicts of Interest

The authors declare no conflicts of interest.

## Supporting information

Supporting information.

## Data Availability

All data generated or analysed during this study are included in this published article (and its Supporting Information Files).
