# Supplementary material for: Delivery of Supported Self‐Management in Primary Care Asthma Reviews: Insights From the IMP2ART Programme
Source: Health Expect. 2024 Nov 12;27(6):e70100. doi: 10.1111/hex.70100 (PMC11555607; doi:10.1111/hex.70100)
Supplement: Supplementary file 1 — Supporting information. [file HEX-27-e70100-s001.docx]

Appendix 1: ALFA toolkit coding framework


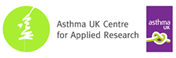

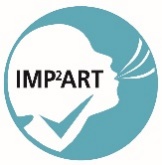


**IMP^2^ART (IMPlementing IMProved Asthma self-management as Routine)**

***Delivery of Supported Self-Management in Asthma Reviews: Insights from the IMP^2^ART Programme***

**Objective 1** – How are self-management tasks prioritised within asthma consultations? (What proportion of time within a consultation is spent on self-management related tasks?). Adaptions of ALFA Toolkit Method 2 (de Lusignan, 2008): a Framework for describing common components of a standard asthma consultation.

**Practice Name: _______________ Professional Name: __________________**

**Mode of Consultation (face-to-face/video/telephone): ___________________**

**Patient/Recording ID:____________ Date of Review:____________________**

| **Grouping** | **Component within review (type of communication or behaviour between both healthcare professional and patient)** | **Time taken (in seconds) to complete the task throughout review** |
| --- | --- | --- |
| **Self-Management** (PRISMS components) | A1: Time spent discussing individual asthma condition and/or its management |  |
|  | A2: Time spent referring to other available asthma resources and services (improving access to services) |  |
|  | A3: Time spent collaboratively reviewing and completing personalised asthma action plan |  |
|  | A4: Time spent discussing attendance to regular reviews |  |
|  | A5: Time spent providing feedback on individual monitored asthma data |  |
|  | A6: Time spent discussing asthma control and possible triggers, practical support with adherence (medication or behavioural) |  |
|  | A7: Time spent providing equipment/discussion of new equipment |  |
|  | A8: Time spent discussing how to access advice or support when needed |  |
|  | A9: Time spent training/rehearsal to communicate with healthcare professionals |  |
|  | A10: Time spent on training/rehearsal for everyday activities |  |
|  | A11: Time spent training/rehearsal for practical self-management activities (e.g., inhaler technique) |  |
|  | A12: Time discussing psychological strategies (problem solving, goal setting, action planning, relaxation techniques etc) |  |
|  | A13: Time discussing individual social support |  |
|  | A14: Time spent discussing lifestyle factors e.g., smoking, diet, exercise |  |
|  | A15: Other: Time spent on screen sharing (discussing something on screen with the patient) |  |
|  | A16: Other: time spent setting the patient agenda for the consultation |  |
|  | A17: Other: Time spent talking about other conditions or multimorbidity |  |
|  | Other Self-management – Please state: |  |
